# Impact of Surgical Mask Placement Over High-Flow Nasal Cannula on Oxygenation Parameters in COVID-19 Patients Experiencing Hypoxemic Respiratory Failure

**DOI:** 10.7759/cureus.75871

**Published:** 2024-12-17

**Authors:** Aadila Coatwala, Mayank Dhir, Sagar Sinha, Dattatray Bhusare

**Affiliations:** 1 Department of General Surgery, K.J. Somaiya Hospital and Research Center, Mumbai, IND; 2 Department of Emergency Medicine, MNR Medical College and Hospital, Hyderabad, IND; 3 Department of Emergency Medicine, MGM Medical College and Hospital, Navi Mumbai, IND

**Keywords:** covid-19, high flow nasal cannula (hfnc), hypoxemia respiratory failure, quick sequential organ failure assessment (qsofa), rox index

## Abstract

Background: During the COVID-19 pandemic, managing respiratory failure in critically ill patients has presented significant challenges. A high-flow nasal cannula (HFNC) has been established as an effective respiratory support modality, offering heated, humidified oxygen at high flow rates. However, concerns persist regarding the potential for aerosol dispersion and the risk of viral transmission, particularly in COVID-19. This study investigates the impact of surgical mask (3-ply surgical mask) placement over HFNC on oxygenation parameters in COVID-19 patients experiencing hypoxemic respiratory failure.

Methods: A retrospective analysis of clinical data from a tertiary medical facility was conducted. The study included 35 patients with confirmed COVID-19 and moderate to severe hypoxemia. Oxygenation indices such as the SpO_2_/FiO_2_ (SF) ratio, flow rate, and the ratio of oxygen saturation index (ROX index) were monitored before and after the application of surgical masks over HFNC. Statistical analyses were performed to compare these parameters before and after surgical mask placement.

Results: The adjunctive use of surgical masks over HFNC significantly improved oxygenation parameters compared to HFNC alone. Despite these improvements, there was no significant change in heart and respiratory rates, quick sequential organ failure assessment (qSOFA) scores, or Glasgow Coma Scale (GCS) levels. Subgroup analysis showed an increase in SF ratio ranging between 5.49% and 6.04% in patients with ROX indices, but these trends were not statistically significant.

Conclusion: This study provides preliminary evidence that surgical masks over HFNC may enhance oxygenation in critically ill COVID-19 patients with hypoxemic respiratory failure. These results underscore the potential importance of infection control measures in respiratory therapy during pandemics and suggest that further investigation in more extensive prospective studies is warranted.

## Introduction

In the context of COVID-19, high-flow nasal cannula (HFNC) and surgical masks are essential tools in managing patients and preventing the spread of the virus. HFNC can be a valuable surgical mask placement for patients with respiratory distress, especially in cases of hypoxemic respiratory failure. However, it is important to note that HFNC might not be suitable for patients with severe respiratory compromise, and more advanced respiratory support, such as mechanical ventilation, may be needed in those cases. HFNC has demonstrated safety as a therapy for individuals with less severe SARS-CoV-2 hARF. However, its effectiveness is still under evaluation [1]. Due to the increased demand for critical care services caused by the COVID-19 pandemic, HFNC, non-invasive ventilation, and continuous positive airway pressure are now used outside intensive care or high-care settings. This presents new issues for healthcare professionals and may put patients at risk [2]. Placing a surgical mask on a patient who is already being treated with an HFNC device increases the oxygen levels of COVID-19 patients admitted to the intensive care unit for severe hypoxemic respiratory failure without any significant adverse effects [3]. The ROX index, which is the ratio of oxygen saturation (SpO_2_) to a fraction of inspired oxygen (FiO_2_) divided by respiratory rate, has been suggested as a means to identify HFNC failure [4]. Another study found that a ROX index cut-off of 4.88, assessed 12 hours after starting HFNC therapy, was linked to a reduced likelihood of requiring intubation [5]. This suggests that the ROX index can help identify patients at a high risk of needing intubation.

Nevertheless, the effectiveness of the ROX index in predicting intubation and the appropriateness of the ROX index cut-off in COVID-19 patients is still uncertain. The SpO_2_/FiO_2_ (SF) ratio, which is related to the PaO_2_/FiO_2_ (PF) ratio [6], can also be used as a prognostic indicator for acute hypoxemic respiratory failure [7]. Significantly, the SF ratio has been identified as a dependable indicator for anticipating the ineffectiveness of HFNC [8] or noninvasive ventilation [9] in real-world medical settings where arterial blood gas (ABG) collection is not easily accessible. In addition, COVID-19 typically exhibits silent hypoxia, which is a situation when the patient does not show any aberrant breathing patterns while having significant oxygen deprivation. Therefore, the respiratory rate may not be a reliable indicator for predicting the failure of HFNC treatment in COVID-19 cases. Consequently, we postulated that surgical masks had a similar predictive capacity to SPO_2_ and ROX index in patients who underwent HFNC with severe COVID-19. Our objective was to assess the alterations in SPO_2_, ROX index, and SF ratio subsequent to the application of a surgical mask over HFNC.

## Materials and methods

Study design and setting

This retrospective analysis was conducted at a hospital, a tertiary healthcare facility, during the second wave of the COVID-19 pandemic in India, from April 2021 to August 2021.

Participants

The study included patients diagnosed with COVID-19 pneumonia who were experiencing hypoxemic respiratory failure and required critical care. Inclusion criteria required patients to be unable to maintain a target SpO_2_ level of 94% with increased oxygen support systems. Exclusion criteria excluded patients who required immediate intubation and those primarily suffering from ventilation-perfusion mismatch rather than diffusion issues. A total of 35 patients met the inclusion criteria and were enrolled in the study, which compared the effectiveness of surgical masks with HFNC. The study did not include a control group, and the impact of wearing surgical masks atop HFNC was analyzed.

Surgical mask placement

Upon admission to the ICU, all patients were connected to a HFNC device (Optiflow TM RT202, Fisher & Paykel, Auckland, New Zealand) and monitored using the Intellivue Patient Monitor MP70 (Philips MedizinSystemeBoeblingen GmbH, Boeblingen, Germany). All COVID-19-positive patients were mandated to wear surgical masks to reduce the spread of airborne germs and aerosols to healthcare workers, as per institutional policy.

Data collection

Continuous monitoring and data recording were conducted from the time of admission after the patient's condition had stabilized, 30 minutes after the mask was placed, and throughout their entire stay. ABG samples were collected via arterial lines to measure oxygenation parameters.

Variables

The primary variables measured included SpO_2_, SpO_2_/FiO_2 _(SF) ratio, and ROX index. Data were consistently captured regularly, maintaining a constant flow rate and FiO_2_ for accuracy.

Statistical analysis

Data are presented as mean ± standard deviation (SD). The normal distribution of differences was verified using Q-Q plots. Paired t-tests were used to compare data collected before and after surgical mask replacement. For repeated measurements, within-factor and between-factor ANOVA with F tests were conducted to analyze SpO_2_, SF ratio, and ROX index. Pairwise differences were tested using the Wilcoxon test with Bonferroni correction to adjust for multiple comparisons. All tests were two-tailed, with a significance level of p < 0.01 considered statistically significant. Statistical analyses were performed using GraphPad Prism version 8.1.2.

Ethical considerations

The study maintained the anonymity and confidentiality of the data, which was used solely for research purposes. Ethical approval was obtained from the Institutional Ethics Committee (IEC) of Mahatma Gandhi Mission Medical College with the approval number DHR-EC/2022/03/03 on March 31, 2022. As this is a retrospective study and it did not require informed consent, the institutional ethics committee approved the same.

## Results

Demographic data of study participants

The average age of the study participants is 56 (SD ± 16), among which males comprised 65% and female participants comprised 35%.

Physiological parameter analysis before and after surgical mask placement

**Figure 1:**
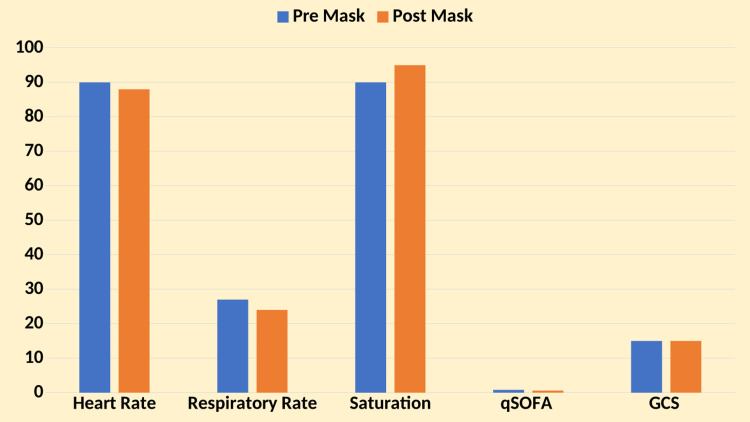
Physiological parameters pre- and post-intervention qSOFA: quick sequential organ failure assessment; GCS: Glasgow Coma Scale

The pre-surgical mask placement means heart rate was 90 beats per minute (bpm) (90 ± 15), and the post-surgical mask placement heart rate decreased to a mean of 88 bpm (88 ± 12), showing a slight but statistically insignificant decrease (p = 0.01). The pre-surgical mask placement means the respiratory rate was 27 breaths per minute (bpm) (27 ± 7), and the post-surgical mask placement respiratory rate decreased to a mean of 24 bpm (24 ± 5), demonstrating a significant reduction (p < 0.05). The pre-surgical mask placement means qSOFA score was 0.8 (0.8 ± 0.4), and the post-surgical mask placement qSOFA score remained almost stable at a mean of 0.6 (0.6 ± 0.4), with no statistically significant difference observed (p = 0.01). The pre-surgical mask placement mean GCS score was 15 (SD ± 0), and the post-surgical mask placement GCS score remained stable like qSOFA to a mean of 15 (SD ± 0), but this was again not statistically significant (p = 0.32). Pre-surgical mask placement mean SpO_2_ was 90% (SD ± 4), and post-surgical mask placement SpO_2_ increased to a mean of 95% (SD ± 0), demonstrating a significant improvement (p < 0.05) (Figure 1).

These findings indicate that surgical mask placement over HFNC was associated with notable improvements in respiratory rate and oxygen saturation. In contrast, heart rate, qSOFA score, and GCS showed trends toward stabilization or improvement, albeit without statistical significance in all parameters except respiratory rate and SpO_2_.

Oxygenation parameters before and after surgical mask placement over HFNC

**Figure 2:**
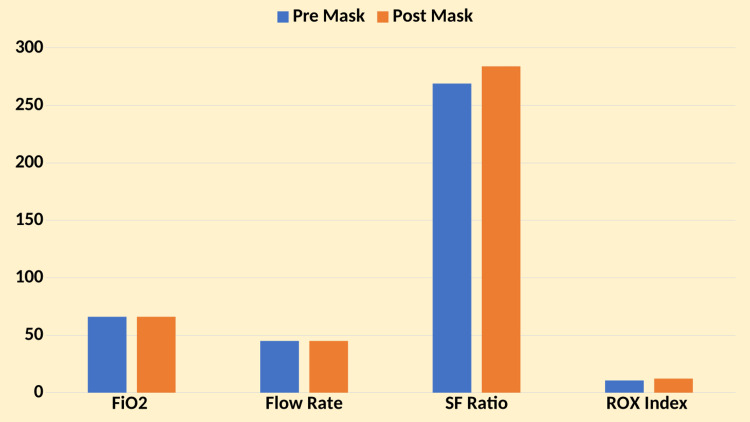
Oxygenation parameters pre- and post-intervention SF ratio: SpO_2_/FiO_2_ ratio; ROX index: ratio of oxygen saturation index

Pre-surgical mask placement mean FiO_2_ was 66 (SD ± 19), and post-surgical mask placement FiO_2_ remained stable at a mean of 66 (SD ± 19), with no statistically significant difference observed (p = not applicable). The pre-surgical mask placement means HFNC flow rate was 45 L/min (SD ± 14), and the post-surgical mask placement flow rate was similar at a mean of 45 L/min (SD ± 14), showing no statistically significant difference (p = not applicable). The pre-surgical mask placement mean SF ratio was 269 (SD ± 184), and the post-surgical mask placement SF ratio increased to a mean of 284 (SD ± 195), demonstrating a significant improvement (p < 0.05). The pre-surgical mask placement mean ROX index was 10.63 (SD ± 7.95), and the post-surgical mask placement ROX index improved to a mean of 12.24 (SD ± 9.03), showing a statistically significant improvement (p < 0.05) (Figure 2).

These findings indicate that surgical mask placement over HFNC was associated with significant improvements in SF ratio and ROX index, suggesting enhanced oxygenation efficiency despite no substantial changes in FiO_2_ or flow rate. The observed improvements in the SF ratio and ROX index highlight the potential benefits of adjunctive surgical mask use in optimizing oxygen delivery and respiratory support in critically ill COVID-19 patients with hypoxemia respiratory failure.

SF ratio subgroup analysis

**Figure 3:**
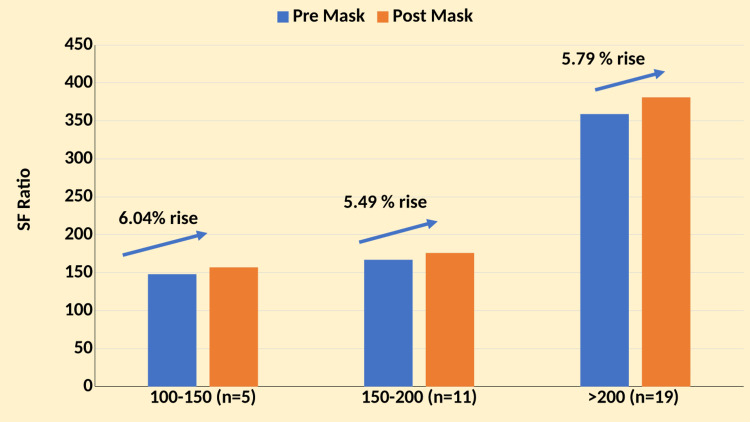
SF ratio subgroup analysis SF: SpO_2_/FiO_2_

A subgroup analysis based on SF ratio categories was conducted to evaluate the impact of surgical mask placement over HFNC on oxygenation parameters among critically ill COVID-19 patients with hypoxemia respiratory failure. The study included 35 patients categorized into three groups based on their SF ratio at baseline: 100-150 (n = 5), 151-200 (n = 11), and >200 (n = 19). Pre-surgical mask placement baseline measurements were compared with post-surgical mask placement values after applying surgical masks over HFNC within each subgroup. The results are summarized as follows (Figure 3).

SF Ratio: 100-150

The pre-surgical mask placement mean SF ratio was 148 (SD ± 3), and the post-surgical mask placement SF ratio increased to a mean of 157 (SD ± 3), showing a trend toward improvement (6.04%) with statistical significance (p < 0.05).

SF Ratio: 151-200

The pre-surgical mask placement mean SF ratio was 167 (SD ± 14), and the post-surgical mask placement SF ratio improved to a mean of 176 (SD ± 15), demonstrating a statistically significant improvement (5.49%, p < 0.05).

SF Ratio: >200

The pre-surgical mask placement mean SF ratio was 359 (SD ± 212), and the post-surgical mask placement SF ratio remained stable at a mean of 381 (SD ± 224), with improvement (5.79%), and a statistically significant difference was observed (p < 0.05).

Across all SF ratio subgroups, the application of surgical masks over HFNC resulted in varied responses, and a combined analysis across all subgroups showed a statistically significant improvement in SF ratio post-surgical mask placement (p < 0.05). These findings suggest that surgical mask placement over HFNC may particularly benefit patients with initial SF ratios between 151 and 200, indicating improved oxygenation efficiency in this subgroup. While trends toward improvement were observed in other subgroups, statistical significance varied. These results underscore the potential role of surgical masks in optimizing oxygenation outcomes among critically ill COVID-19 patients receiving HFNC therapy.

ROX index subgroup analysis

**Figure 4:**
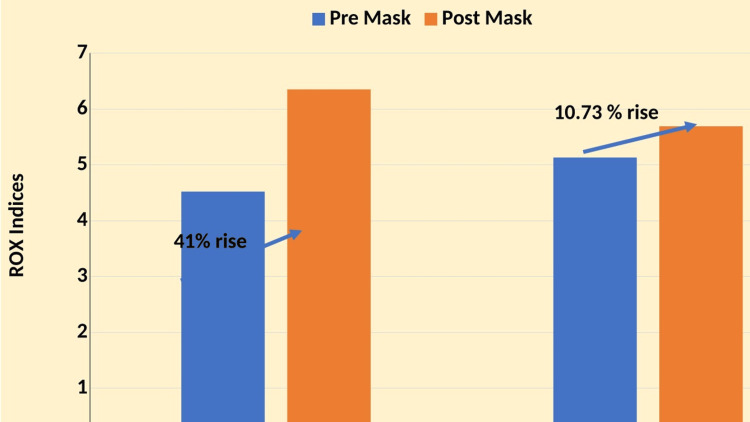
ROX index subgroup analysis ROX: ratio of oxygen saturation

A subgroup analysis included 35 patients categorized into two groups based on their ROX index at baseline: 3.85-4.88 (n = 3) and >4.88 (n = 32). Pre-surgical mask placement baseline measurements were compared with post-surgical mask placement values after applying surgical masks over HFNC within each subgroup (Figure 4).

ROX Index: 3.85-4.88

The pre-surgical mask placement mean ROX index was 4.52 (SD ± 0.15), and the post-surgical mask placement ROX index improved to a mean of 6.35 (SD ± 1.65), showing a trend toward improvement (41%) but without statistical significance (p = 0.2).

ROX Index: >4.88

The pre-surgical mask placement mean ROX index was 5.13 (SD ± 8.08), and the post-surgical mask placement ROX index also showed a rise (10.73%) at a mean of 5.69 (SD ± 9.25), with a statistically significant difference observed (p < 0.05).

Across both ROX index subgroups, the application of surgical masks over HFNC resulted in varied responses, and a combined analysis of all subgroups showed an overall improvement in ROX index post-surgical mask placement. These findings suggest that while trends toward improvement in the ROX index were observed in both subgroups, statistical significance was only reached when analyzing all patients together. This indicates that surgical mask placement over HFNC may contribute to optimizing oxygenation outcomes across different initial ROX index categories among critically ill COVID-19 patients. Further studies with larger sample sizes are warranted to validate these findings and explore additional clinical implications.

## Discussion

The study analyzed physiological parameters before and after surgical mask placement over HFNC in critically ill COVID-19 patients with hypoxemic respiratory failure. Results showed that with pre-surgical mask placement, heart rate decreased to 88 bpm, respiratory rate decreased to 24 bpm, qSOFA score remained almost stable, GCS score remained stable, and SpO_2_ increased to 95%. Oxygenation parameters showed significant improvements in the SF ratio and ROX index, suggesting enhanced oxygenation efficiency despite no substantial changes in FiO_2_ or flow rate. Our result supports previous research showing a significant improvement in oxygenation among hypoxemic COVID-19 patients admitted to the ICU when a surgical mask is placed on the patient's face [3,10]. The observed improvement in oxygenation parameters could be explained not only by an increased oxygen concentration in front of the mask but also by a decrease of room air entrainment that is known to dilute the gas mixture with less inspired O_2_ concentration [10]. A subgroup analysis was conducted to evaluate the impact of surgical mask placement over HFNC on oxygenation parameters among critically ill COVID-19 patients with hypoxemic respiratory failure. The results showed a statistically significant improvement in SF ratio post-surgical mask placement, particularly for patients with initial SF ratios between 151200. The application of surgical masks over HFNC resulted in varied responses across all SF ratio subgroups, suggesting the potential role of surgical masks in optimizing oxygenation outcomes among critically ill COVID-19 patients receiving HFNC therapy. These findings imply that the SF ratio might serve as an effective screening tool for hypoxemia in the emergency department, as reported by a study [11]. According to their multivariate regression study, a study found that the SF ratio is a strong predictor of intubation risk in hypoxemic patients with COVID-19 [12]. Our investigation found that the SF ratio had a 7.3% rise following the provision of surgical masks across all ages and genders. A separate investigation has recorded the ROX index (at six hours) and the SF and PF ratios (at six hours) as reliable indicators of HFNC failure [13]. Due to the isolation of COVID-19 patients in a confined space and the restricted number of healthcare staff, regular ABG analysis is not feasible. In addition, the objective monitoring of respiratory rate may provide challenges due to the inadequate accuracy of measurements conducted by healthcare personnel [13,14]. Conversely, the SF ratio may be determined using objective pulse oximetry and FiO_2_ measurements. Hence, it can be proposed that SF ratios serve as a valuable tool for anticipating the necessity of surgical masks in COVID-19 patients.

The SF ratio after the commencement of HFNC was a reliable predictor of HFNC failure as demonstrated by Kim et al. (2022) [15]. They reported that the SF ratio may serve as an effective prognostic indicator for anticipating intubation in COVID-19 patients undergoing HFNC. Kansal et al. (2022) suggested a modified dynamic indicator (Delta POX-HR) may enhance early and precise prediction of HFNC outcomes compared to the ROX index in ARF patients with diverse etiologies [16]. Choi et al. (2022) conducted a study indicating that the ROX index, ROX-HR index, and SF ratio are promising tools for the early prediction of therapy success or failure in patients commencing HFNC for acute hypoxemic respiratory failure [17]. In ED patients with COVID-19, Ruangsomboon et al. (2023) also reported that the SF ratio was a better predictor of HFNC success than the ROX and modified ROX indices [18].

ROX index subgroup analysis showed varied responses across both subgroups, with trends toward improvement observed in both subgroups. However, statistical significance was only reached when all patients were analyzed together. This indicates that surgical mask placement over HFNC may contribute to optimizing oxygenation outcomes across different initial ROX index categories among critically ill COVID-19 patients. Further studies with larger sample sizes are warranted to validate these findings and explore additional clinical implications. Several studies have considered ROX and mROX indices as valuable tools in predicting intubation in COVID-19 patients treated with high-flow nasal oxygen in the ICU [19-21]. Although our study did not present ROX index data at 1 hour, 2 hours, 4 hours, 6 hours, 8 hours, 12 hours, and 24 hours after HFNC initiation and mask replacement, it was significantly higher than the previous studies.

HFNC therapy delivers a higher flow of humidified and heated oxygen through nasal prongs, which can help improve oxygenation and reduce the need for invasive mechanical ventilation in some cases. Using a surgical mask with HFNC may serve several purposes: aerosol containment (COVID-19 is primarily transmitted through respiratory droplets. The use of a surgical mask can help contain respiratory droplets and reduce the risk of spreading the virus to healthcare workers and other patients) and oxygen enrichment (the placement of a surgical mask over the patient's nose and mouth may help to increase the concentration of oxygen delivered through the HFNC system, potentially improving oxygenation). It is important to note that the effectiveness of this approach may depend on various factors, including the severity of the patient's respiratory failure, the stage of the disease, and individual patient characteristics, including age and sex. Surgical masks are typically designed to protect the wearer and the surrounding individuals from respiratory droplets, and they play a crucial role in preventing the spread of infectious diseases like COVID-19 [22]. Different types of surgical masks are available, and their use by COVID-19 patients can vary based on the situation and recommendations from healthcare authorities. A recent study has demonstrated that a non-rebreather mask and a low-flow nasal cannula are more effective than HFNC in increasing oxygen levels in patients with COVID-19-related respiratory failure characterized by low oxygen levels [23].

Study limitations

The retrospective nature of the study does not allow causality to be established, and there may be unknown confounders influencing the observational data. The sample size is small at 35 patients, and the results cannot be generalized and have limited statistical power to perform subgroup analyses. As it is conducted in a single tertiary care hospital, the results may not be generalizable to other healthcare settings where the patient population or practice may be different. The lack of randomization introduces the risk of selection bias because patients were not randomized to receive a surgical mask over HFNC. In addition, the study only covered oxygenation parameters, namely the SF ratio, flow rate, and ROX index, and did not consider other relevant clinical outcomes, such as ICU stay duration, requirements for mechanical ventilation, or mortality. There were no assessments of long-term effects and sustainability during this short-term monitoring period, and potential measurement bias due to reliance on existing medical records may exist. Also, the absence of a control group receiving HFNC without a surgical mask cannot be used to determine which improvements are attributable to the surgical mask placement. Clinical management and infection control practices also were variable, which complicated interpreting the findings.

## Conclusions

The study revealed that placing surgical masks over HFNC significantly improved respiratory rate, oxygen saturation, SF ratio, and ROX index. Specifically, patients with initial SF ratios between 151 and 200 showed notable enhancement in oxygenation efficiency. Across all SF ratio subgroups, the application of surgical masks over HFNC led to statistically significant improvements in the SF ratio. Similarly, across ROX index subgroups, there was an overall enhancement in the ROX index following the surgical mask placement. These findings underscore the potential advantages of incorporating surgical masks to optimize oxygen delivery and respiratory support in critically ill COVID-19 patients experiencing hypoxemic respiratory failure.
